# Medical Items and News of Interest to the Physician

**Published:** 1881-10

**Authors:** 


### Medical Items and News,

### For the Use of Physicians.

CULLED FROM THE STANDARD MEDICAL JOURNALS OF THE WORLD.

**Chilblains.**

Acid. carbol, ʒ j.

Tinct. iodinii, ʒ ij.

Acid. tannici, ʒ ij.

Ceret. simplicis, ℥ iv.

M. Sig. Ointment.—*Dr. Bartholow, Medical Brief.*

**Diarrhœa.**

Tr. opii, ʒ ii.

Tr. capsicum, ʒ iv.

Tr. camphor, ʒ iv.

Syr. rhei. arom, ℥ iss.

M. Sig. Teaspoonful pro re nata.

**Cholera Mixture.**

Sul. ether.
Aqua. ammonia.
Tr. opii.
Tr. capsicum, āā ʒ iv.

M. Sig. Teaspoonful pro re nata.

**Coughs and Night Sweats of Phthisis.**

Acid. gallic, gr. viii.
Ext. cannabis indica, gr. ss.
Morphia hydrochlorate, gr. ¼.
Confection rosa, q. s. ad.

Pill. No. 2. Sig. To be taken at bedtime.

**Cholera Infantum.**

Syr. rhei. arom, ℥ ii.

Aqua calcis, ʒ iv.

Aqua menth pip., ʒ ii.

M. Teaspoonful every hour until it operates. If discharges are light or clay-colored, give powder of hyg. cum creta every three hours until they change.

**Cystitis.**

Dr. Skene, of Brooklyn, gives the following as almost a specific in cystitis, especially in the earlier stages, afford rapid and lasting relief:

℞
Acidi benzoici.
Sodii biboratis, āā gr. x.
Inf. buchu, ℥ ij.

M. Sig—This quantity to be taken three or four times a day. The diet should be regulated, and the skin and bowels kept in an active condition.—*Dr. Skene, Brooklyn, Ther. Gaz.*

**Inflammation of the Mammæ.**

In the Philadelphia *Medical and Surgical Reporter*, Dr. H. B. Stehman claims good results, in mammary inflammation, from the leaves of jaborandi applied to the breast in the form of a poultice. It always cuts short the disease if used before suppuration commences. It is also of great use in mumps and buboes, if applied early.

**Acute Catarrh.**

Tinct. iodinii, ℥ ss.
Acid. carbol, ʒ j.

M. Sig. Place a small, wide-mouthed bottle, containing a moistened sponge, in a vessel of hot water ; drop five to ten drops of the solution on the sponge, and as the iodine vapor ascends with the vapor of the water, inhale it. —*Bartholow. Monthly Review of Medicine and Pharmacy.*

**Chronic Malarial Poisoning.**

Acidi arseniosi, gr. ij.
Strychniæ sulph, gr. ij.
Quiniæ sulphatis, gr. lxxx.
Manganesii sulphatis, gr. xl.
Ferri sulphatis exsic, gr. xxx.
Ext. calumbæ, q. s.
Olei eucalypt. ether, ♏. xx.

M. ft. pil. No. lxxx. S.—Take two pills three times daily at meals.—*Medical Herald.*

**Summer Dysentery of Children.**

On this subject, Dr. W. W. S. Watson, of New Holland, Ill., says in the *Mich. Med. News*, July 11th, 1881, that the first indication is to thoroughly rid the alimentary canal of irritating ingesta. This is most promptly affected by means of a saline purge, which, in addition to its action in this direction, also depletes the inflamed tissue through the exosmosis of serum which it causes. Castor oil is preferred by some; it is blander in its action, but adds nothing to its purgative action. After this thorough cleansing of the canal the indication is to check the discharge. For this purpose he relies on opium and ant-acids, after the following formula, modified, of course, according to age and other circumstances:—

℞

Syr. rhei, ℥ iss.

Spt. ammoniæ arom., ʒ ss.

Spt. camphoræ, ʒ ss.

Tr. opii, ʒ j.

M. Sig.—A teaspoonful every three hours to a child eight years of age.

**Facial Erysipelas.**

Quiniæ sulph. ʒ ss.

M. Belladonnæ ext, gr. iij.

Ft. pil. No. X.

Sig.—One every four to six hours.—*Dr. Bartholow.—Michigan Med. News.*

---

**Cholera Mixture.**

℞

Tinct. opium.

" camphor.

" capsicum.

" rhubarb.

Essence of peppermint, āā ℥ ii

M. Sig—A teaspoonful in water after each evacuation of the bowels.

---

**Quebracho.**

The results of recent experience with this drug in Bellevue Hospital have been confirmatory of its value in dyspnœa in all its forms. The fluid extract, in doses of from twenty to sixty drops, every hour or two as called for by the symptoms, has been found useful in our hands also, without regard to the exciting cause of the dyspnœa.—*Independent Practitioner.*

---

**Eclampsia.**

M. Sanger publishes three cases of eclampsia treated by subcutaneous injection of pilocarpine in two centigramme doses. The crisis seemed to yield, but the patient experienced suffocative symptoms from inability to swallow the saliva. Two died. Pilocarpine is a very dangerous remedy in these cases, as the coma stops the reflex actions necessary for the swallowing of the saliva.—*La Presse Medicale Belge.*

---

**Itch.**

Sponge the whole body, night and morning with a solution of chloride of lime, or use an ointment composed of

℞

Liquid Styrax, ʒ jss

Lard, ʒ ij.

Mix and strain. This applications will kill the acarus without irritating the skin.

Another formula is

℞

Olive oil, ʒ j.

Alcohol, ʒ ij.

Liquid styrax, ʒ j.

Mix. The mixture to be thoroughly rubbed over the whole surface of the body, and to be repeated upon the following day. A general bath on the third day will remove the lotion and the dead acari.

**Erysipelas.**

In a recent number of the *Archives of Dermatology*, Dr. L. Heppel, of this city, makes known a new abortive treatment of erysipelas which he has so far known to be successful in seven cases. It consists in "brushing the boundary line and the parts extending a finger's width on either side of it, with a ten per cent. alcoholic solution of carbolic acid until the integument thus painted shows a decided discoloration." An agreeable sensation is said to be experienced at the points of application.

---

**Freckles.**

The following formula is said to be efficacious for the removal of tan and freckles;

℞
Hyd. bichlor, grs. vj.
Acid, mur. dil., ʒ j.
Aquæ, ℥ jv.
Alcohol, ℥ ij.
Aq. rosæ, ℥ ij.
Glycerine, ʒ j.

M. Sig—Apply at night and wash off with soap in the morning.—*Canada Lancet.*

---

**Bilious Colic.**

A correspondent in Iowa speaks highly of the the fluid extract of this indigenous plant, Dioscorea Villosa, and he relates a very severe case in which a teaspoonful dose gave immediate relief. The wild yam has long had a reputation as a "colic root." It is mentioned in the appendix of the United States Dispensatory, but as Prof. Stille remarks, in the last edition of the National Dispensatory, "Time has not added to our knowledge of its properties." He thinks its active principle is allied to ipecac.—*Med. and Surgical Reporter.*

---

**Croupous Pneumonia.**

Iodine or iodide of potassium is, according to Schwarz (*Deutche Med. Woch.*), a specific in simple uncomplicated croupous pneumonia. If given during the first twenty-four to thirty-six hours from the onset of the initial rigor, it will arrest the further progress of the disease. In illustration of this view, ten cases are recorded in which the crisis occurred before the end of the second stage, and in one case at the end of the first day. The following are the formulæ adopted by Schwarz: Tincture of iodine, five drops; water, 120 grammes (4 ounces); one tablespoonful hourly. Iodide, 1½ grammes (22 grains); simple syrup, 30 grammes (1 ounce); water, 130 grammes (4 ounces); one tablespoonful hourly—*London Med. Record.*

**Eclampsia.**

M. Guyot related to the Society Medicale des Hopitaux, that a child, aged eleven and a half years, was seized with an attack of eclampsia on the twenty-second day of an attack of scarlatina complicated with albuminuria. He was in a state of coma, and near death, when M. Guyot bled him to the extent of 300 grams and gave him two enemata of chloral. The child recovered rapidly.—*Med. and Surg. Reporter.*

**Asthma.**

Grindelia Robusta is being revived again as a remedy for asthma. Where the fluid extract is pure, it certainly often is of great service in this wretched malady. It should be given in dram or half dram doses hourly or half hourly during the paroxysms, and less frequently after relief is obtained; but should be continued some days. The fluid extract is also often of service in the poison-oak eruption applied externally. —*American Practitioner.*

**Sore Nipples.**

When cracked nipples are not caused by constitutional disease, they should be freely washed with tincture of benzoin. Under this treatment they will generally heal in from five to ten days. The benzoin forms a varnish over the surface of the cracks, and this protects them during the act of nursing. The great advantage of the treatment is, that in no wise interferes with lactation.—*E. M. Jour., St. Louis.*

**Hysteria and Epilepsy.**

M. M. Bourneville and D'Olier, have instituted a series of researches upon the physiological and therapeutic action of ethyl bromide in cases of epilepsy and hysteria. The conclusions at which they have arrived are as follows:—(1). The dilatation of the pupil, which may occur at the commencement of the inhalation of the drug is not a constant phenomenon. (2). Complete muscular relaxation is exceptional. (3). The anæsthesia produced varies greatly in degree in different individuals. (4). The temperature, secretions, and general state do not undergo any modification. (5). The pulse and respiration are slightly quickened. (6). A more or less well-marked trembling may occur in the limbs during inhalation, but it is only transient. (7). Hysterical fits are usually arrested with ease by the administration of ethyl bromide. (8). The onset of an epileptic attack may sometimes be arrested by giving the remedy from the tonic stage; but the inhalation of the drug is more commonly inoperative. (9) In epilepsy the regular use of bromide of ethyl, administered in the form of vapor every day for a period of four to eight weeks, diminishes to a very great extent the frequency of the fits. —*Le Progres Medical.*

**Growth of Hair.**

The *Union Pharmaceutique* last year published a note to the effect that a German physician had been struck with the curious production of hair in places where he had applied pilocarpine in hypodermic injection. A pharmacist having read that note, writes to say that he has found a mixture of tincture of cinchona and arsenic in which jaborandi leaves have been macerated produce excellent results.

**Asthmatic Paroxism.**

Tinct. lobeliæ, ℥ j.

Ammon. iodidi, ʒ ij.

Ammon. bromidi, ʒ iij.

Syr. tolutan, ʒ iij.

M.

Sig.—A teaspoonful every one, two, three, or four hours.—*Dr. Roberts Bartholow.*

Of this prescription Dr. Bartholow says: "It gives relief in a few minutes, and sometimes the relief is permanent."—*Michigan Med. News*

**Chronic Rheumatism.**

Alterative medicines are often of the greatest value in chronic rheumatic affections. Where only one or two joints are affected and there is considerable effusion, iodide of potash and bichloride of mercury in compound syrup of sarsaparilla, or bichloride of mercury in compound infusion of gentian, is often used. The dose of the mercurial being $\frac{1}{60}$ to $\frac{1}{40}$ of a grain. When a number of joints are involved, especially where there are gouty complications, the following is used:

℞

Pulv. guaiaci, ʒ i.

Vin. colch. rad., ʒ ij–iij.

Potass. iodid, ʒ i.

Pulv. acaciæ, q. s.

Sp. lavand. comp., ℥ ss.

Aq. cinnam, q. s., ad ℥ vj.

M. Ft. sol. S. Dessertspoonful three times a day in water.

Other salts of potash may sometimes be used with advantage instead of the iodide, and lithia is also of marked value in some cases.—*St. Louis Courier of Med. & Surg.*

**Acute Bronchitis.**

Vini ipecacuanhæ, ʒ ij.
Liq. potassii citratis, ℥ iv.
Tinct. opii camphoratæ,
M. Syrupi acaciæ, āā ℥ j.

Sig.—A tablespoonful thrice daily in the first stage of ordinary acute bronchitis.—*Dr. Da Costa.*

This union of the sedative effects of opium with the excito-secretory action of the ipecacuanha on the congested mucuos membrane has been found very serviceable.—*Michigan Med. News.*

---

**Summer Diarrhœa of Children.**

℞

Bismuth subnitrate, ʒ i.

Pepsinæ sacch, ʒ ss.

Zinci oxidi, gr. vi.

M. Ft. pulv. No. xii. One powder every four hours; or—

℞
Plumbi acetat, gr. viii.
Acid acet, gtt. vi.
Tinct. opii deod, gtt. iv.
Aquæ destillatæ, ℥ i.

M. Sig.—A teaspoonful every two, three or four hours for a child two or three years of age.—*Dr. Bartholow.—Mich. Med. News.*

---

**Rhus Aromatica.**

On reading the remarks of Dr. F. T. McClanahan on rhus aromatica and its use in diabetes, I resolved to give it a trial if a proper case presented itself in my practice. Accordingly, on April 4, 1880, the case wished for arrived. Case—Geo. B., æt. 40 years, unmarried; urine loaded with sugar; on applying Trommers' test, specific gravity 1030°; pale and cadaverous looking. Also, in addition to the diabetic condition, was troubled with a constant dribbling of urine, which rendered him most miserable. Gave him fluid ext. rhus aromatica in the following prescription: ℞. Rhus aromatica, ℥ j; glycerine, ℥ iij. M. Sig. Teaspoonful three times a day.

He began to mend at once, and now, on the 11th of December, is as well as he ever was in his life. He can do a good day's work, whereas he could not work at all when he came under my charge. I am convinced that in rhus aromatica we have one of the remedies that is very efficacious in the diseases for which it is recommended.—E. W. M'CORD, M. D., Rock Rapids, Iowa.—*Therapeutic Gazette.*

**Prolapsus Ani.**

R. Eichler, M. D., in *Wesoern Lancet*, says: A boy five years of age came under my treatment, suffering from prolapsus ani of two years standing. The gut came out to the extent of two and a half inches after each passage. My treatment at first was of a routine kind—cold effusions, cauterizations with nitrate of silver, tincture of iron, etc. The bowel persisted in coming down at every passage. As a last resort, I tried an ergotine suppository.

℞
Ergotine, gr. ij.
But. cocoa, q. s.
M. Ft. Suppos, no. j,

One after each passage.

The effect of the remedy has been magical, as after the use of a few of the suppositories, there has been no return of the condition, and the case is cured.

---

**Constipation.**

We read in the Paris Medicale that, Mr. Noel Gueneau de Mussey proposes using Psylium or Sarragota seed, besides white mustard seed, the use of which is excellent, or flax seed in the natural state.

Psylium is a species of plantain, commonly called Fleawort, because of the appearance of its seeds, which are quite small and very mucilaginous. A teaspoonful in half a glass of water is taken before dinner. He says that with a number of persons this method has proven as successful as with the Spanish lady from whom he obtained it. In other cases, however, he was obliged to alternate with more powerful laxatives, such as aloes or rhubard, so as to keep up the effects. It is probable that psylium seed, like others of its kind, is not persistent in its effects, although in a number of cases it seems to have been so.—*Med. and Surg. Reporter.*

---

**Sub-Acute Pleurisy.**

Potas. acet.
Inf. digital, āā dr. ij–iv.

Sig.—This amount each day: or

Pulv. digital,
Pulv. scillæ mar,
Hydrarg. chlo. mit, āā gr. x.
M. Et. ft. pil. No. X.

One pill thrice daily.—*Dr. Alonzo Clark.*

The indications for treatment are to subdue the inflammation and promote absorption of the effused fluid. Dr. Clark, to accomplish the first of these effects, uses blisters, three being usually sufficient, selecting three spots and applying only one blister at a time, the second and third not being placed in position until the spot of former application has healed. As a diuretic he uses potassium iodide grs. xxx a day; if this fail to diminish the fluid he has recourse to the above formulæ. If constitutional effects of mercury declare themselves he returns again to the potassii iodidi. Dr. Clark uses other means, as purgatives, vapor baths, and mild counter-irritants, when he thinks them indicated, but having exhausted all medical means without effect (as is sometimes the case) he either resorts to the trocar or does nothing. —*Michigan Med. News.*

---

**Gout.**

Surgeon Austin Meldon, writing upon the pathology and treatment of gout, draws the following conclusions:—(1). The presence of uric acid and soda in the blood is not the sole cause of gout. (2). Want of exercise and animal diet will produce an accumulation of uric acid in the blood. (3). Uric acid and soda must exist in the blood before the disease can be produced. (4). There must be depression of the nervous system to cause an attack of gout. (5). Depression of the nervous system causes an union between uric acid and soda. (6). When an attack of gout has passed away, it does not necessarily follow that the uric acid has disappeared from the blood. (7). Uric acid may exist in considerable quantities and for any length of time without causing gout. (8). The use of nerve tonics—as quinine, strychnia and caffein, and such like—as well as the inhalation of oxygen and the use of electricity, are of much service in the treatment of the disease—*The British Medical Journal.*

---

**Tetanus.**

The case of a boy, aged 11, is related in the *Lancet.* Tetanus followed a lacerated wound of the heel. Chloral and belladonna in full doses were first tried, but the patient became worse, and was presently unable to swallow his medicine, convulsive attacks being brought on by each attempt. Subcutaneous injections of morphia also caused convulsions and did no good. Gelatine lamels, containing one-sixtieth of a grain of extract of Calabar bean in each, were next obtained. One was given every four hours, being slipped between the teeth and allowed gradually to dissolve in the mouth. Improvement was noted two days later. The lamels were continued for seven days, by which time the boy was convalescing satisfactorily. He has since completely recovered. The case was an extremely unfavorable one at the time when the administration of Calabar bean was commenced. The boy's age, and his ability to take plenty of nourishment throughout his illness, were of course greatly in his favor, but the result affords a fair presumption that the drug had a beneficial effect. It, therefore, deserves a further trial (in the form of gelatine lamels) in similar cases.

---

**Asthma.**

The *New York Medical Journal* publishes three cases of asthma treated with inhalations of iodide of ethyl, with remarkable benefit. They occurred in Dr. R. M. Lawrence's service at the Boston Dispensary. Following the cases are some remarks by Dr. Lawrence, in which he says of the iodide of ethyl: "Its speedy absorption into the blood, its antispasmodic quality, and prompt reflex stimulation of the respiratory muscles, may reasonably account for its beneficial action in the asthmatic paroxysm, while its power of liquefying and detaching accumulations of mucus sufficiently explains its curative influence in chronic bronchitis * * Experience has confirmed my faith in its medical worth in a large majority of cases of labored respiration (whether due to bronchial spasm or to increased mucuous secretion), and also in certain obstinate cases of dyspnœa, and due to organic pulmonary or cardiac lesions, where other remedies may have proved inefficient. In a small minority of cases it has failed to afford relief." He does not recommend it as a substitute for internal medication, but rather as an adjunct thereto.

---

**Diphtheria.**

M. Constantin Paul ends his review of the methods of the treatment of diphtheria, brought before the Society de Therapeutique during the year 1881, in the following terms:

"Diphtheria and diphtheritic poisoning still remain one of the pre-occupations of therapeutics. This year, like the preceding, the results of many new experiments undertaken by members of the society, have been laid before you.

"First, the treatment of the diphtheritic patches by the bromide of potash, by M. Peyraud. M. Cadet de Gassicourt, who repeated conscientiously the treatment of our colleague, has shown, in a very interesting report, that bromide of potassium does not constitute, any more than chlorate of potash, or cubebs, a medication sufficiently efficacious to warrant the belief that we are at last able to control this scourge which has proven so redoubtable for young children and those who nurse them.

"The same must be said of the treatment of M. Brame, cauterization with the iodide of silver; of that proposed by M. Perati, cauterization with carbolic acid and camphor; of that of M. Bergeron, with fluorhydric acid, which had as advantage, that it might be applied almost at distance, without demanding the docility of the child, so difficult to obtain for applications to the throat. Will we be more fortunate with pure carbolic acid, which has been recently proposed by M. Bournonville ?

"It is necessary to say this year, as we concluded last year, that the best treatment in diphtheria is to sustain the forces of the patient by stimulants, particularly alcoholic stimulants; to disembarrass the throat of false membranes by removing them mechanically, when possible; and to perform tracheotomy, in case of obstruction, if the strength of the subject permits.

"Chlorate of potash, cubebs, carbolic acid, camphor, fluorhydric acid, lime water, and tartaric acid, are of use in diphtheria without toxic symptoms, but are insufficient remedies."—*Med. and Surg. Reporter.*

---

**Restoring the Heart's Action.**

I do not remember what induced me to kill a mouse by a blow upon the head, and rip it open to see the heart beat. It did not. I pricked it with a needle, and set it agoing. It stopped after a time. Then I gave it a second prick, and a few pulsations were distinctly seen. When I was in petticoats my father was sent for to see a girl in a fit. He was out, and when he came home he was informed of the fact. "How long ago, and any second message ?" Being told, he thought he need not go. My mother suggested he "ought to go," which he did. He found the girl dressed in her grave clothes and laid out upon a linen-covered table. He examined her and found some warmth over the heart. He ordered hot water to be brought—not scalding hot—and poured it into a jug, tore her shroud open, stood on a chair, and poured a continuous stream of hot water until the throbbings of the heart were distinctly seen. That girl was the mother of several children before I left Scotland in 1848. My mother used to laugh and take her share of the credit of her restoration to life.

An old man here, Robert Robinson, several years before his death took a fit, and apparently expired upon the floor, where he was lying pulseless and breathless. The heart had ceased to beat, and I was told that "he was beyond any doctors's power now." I felt some warmth over the heart, and tried my father's remedy, and to the wonder of spectators the septuagenarian revived and lived several years afterward. Hot water can easily be obtained, and no one can object to such an experiment.—J. C. REID, M. D., *in British Medical Journal.*

---

**Billiary Calculi.**

In the Bulletin de Therapeutique, Prof. Bouchardt gives the following rules: 1st, Abstain from bread, eggs, nitrogenized aliments in excess, tomatoes, strong liquors, from fish, oysters etc., from old cheese. Instead of bread make use of potatoes, and take ordinary vegetables containing potash in preference to those containing soda. Make an indirect alkaline medication by using fruits containing malates and and citrates. 2d, Keep the bowels in a soluble condition, taking a tablespoonful of tartrate of potash or soda, or of sulphate of soda in lemonade, orangeade, each morning. 3d, Moderate exercise. 4th, Render the cutaneous secretions more active by frictions, etc. One two three baths weekly, containing:

℞

Potass. carb., ʒ iij.

Ol. lavand., ʒ ss.

Tr. benzoin, ʒ j, followed by prolonged frictions and massage. 5th, (a) In order to induce expulsion of the calculi, small doses of spr. terebinth, and of ether in capsules, either at meals or preferably, between meals. (b) To prevent the formation of calculi, for ten days, before the morning and evening meal, a pill containing one decigram (gr. jss) of tartrate of potash and lithia; during the ten days following:

℞

Potass. acetat., ʒv.

Syr. sarsæ comp., ℥ xij.

Sig.—Tablespoonful morning and evening.

Then, for a ten days, a quart of water daily, containing ten grams (℥ ijss) of Rochelle salts.—*Med. and Surg. Reporter.*

**Diphtheria.**

Dr. George Guttman, of Cronstadt, claims to have had good results from pilocarpia in the treatment of diphtheria. In an experience of a year and a half, during which a large number of cases, both severe and mild, were treated, his success has been very encouraging. The drug induced abundant secretion from the mucous membrane of the throat and fauces, which brought away the false membrane. No injurious inflammation was excited, nor was there any marked tendency to depression. When symptoms of depression were observed, he alternated the medicine with stimulants in some form. The following are his formulæ:

*For Children:*

℞

Pilocarpiæ mur, gr. $\frac{1}{8}$—$\frac{2}{8}$.

Pepsinæ, gr. i—j$\frac{1}{4}$.

Acid. hydrochlor, gtt. ij.

Aq. dest, ℥ ijss.

M. Sig. A teaspoonful hourly.

*For Adults:*

℞

Pilocarpiæ mur., gr. ss—j.
Pepsinæ, gr. xxx.
Acid. hydochlor, gtt. iij.
Aquæ dest., ℥ viij.

Sig. A tablespoonful hourly.—*Louisville Med. News.*

---

**Affections of the Skin.**

Prof. Kaposi read a paper at Vienna Medical Society, in which he stated that he had employed this substance (the B naphtha oil, largely used by color makers) as an advantageous substitute for tar, being as useful, while not producing the same disagreeable effects as regards color, smell, or spoiling linen. Hitherto he has used it only externally, but from its rapid absorption and excretion it may be used, probably, with good effect internally. How far this is the case, and to what disease it is especially applicable, further trial must prove. At present speaking, from seventy-six cases, Kaposi has found it a useful remedy. Thus, in scabies, a ten per cent. simple ointment followed by a compound ointment of ten parts of naphtha oil, fifty of green soap, and one hundred of lard will effect a cure in two applications. In eczema as with tar, the exact time of application is difficult to choose, but if this be well chosen, the itching is allayed and a slow desquamation occurs. In psoriasis a ten per cent. ointment produces the same effect as a chrysarobin ointment, without the discoloration caused by this. The ointment has also been used with good effect in ichthyosis, seborrhœa, and pityriasis versicolor; but it is no avail in lupus vulgaris; while a case of lupus erythematosus was advantageously treated. The naphtha oil is easily soluble in alcohol and in oils or fats, but water requires equal parts of alcohol to be added to effect a solution.—*Med. and Surg. Reporter.*

---

**Burns.**

Dr. Fuller, of Neukirchen, a mining district in which burns caused by explosions or fire damp, or powder, are frequent, has adopted a mode of treatment of burns by thymol, which he has found attended by most favorable results. Each patient, as soon as admitted to the hospital, receives a warm bath. The burnt surface and its surroundings are then washed with an aqueous solution of thymol (1 to 1,000) followed by the application of thymol spray for several minutes. The blisters are not disturbed, but are handled with extreme care. The raw surface is then painted with a one per cent. thymolized linseed oil. The patient is then laid on a waterproof mattress, the temperature of the room being kept comfortably warm. Particles of coal or other foreign matter, if not too minute, are, as a matter of course, at once removed. It is often very difficult to so lay the patient that the burned places are relieved of pressure, and it is frequently necessary to allow him to remain in a sitting posture, sometimes for several days, with support for the chin, or even *a la vache*, by suspending him by means of wide strips of muslin, passing under the chest or abdomen—the strips being fasten, above. The application of thymol should at first be repeated every ten minutes; and as it relieves pain very remarkably, the patients themselves call for it. For the purpose large soft-haired paint-brushes are used. At first the oil is absorbed somewhat rapidly, and as soon as this has occurred a sensation of intense burning follows. The applications are gradually made less frequently; as an indication of their necessity, the appearance of the skin is sufficient. As soon as the oil is entirely absorbed, it should be replaced by a fresh portion, as it is important to prevent contact with the air. During the first few days the thymol spray is also applied as often as possible, which does much toward alleviating the pain.—*Lond. Med. Record.*

